# Single Atom Alloys Segregation in the Presence of Ligands

**DOI:** 10.1021/acs.jpcc.3c05827

**Published:** 2023-11-13

**Authors:** Maya Salem, Dennis J. Loevlie, Giannis Mpourmpakis

**Affiliations:** Department of Chemical and Petroleum Engineering, University of Pittsburgh, Pittsburgh, Pennsylvania 15261, United States

## Abstract

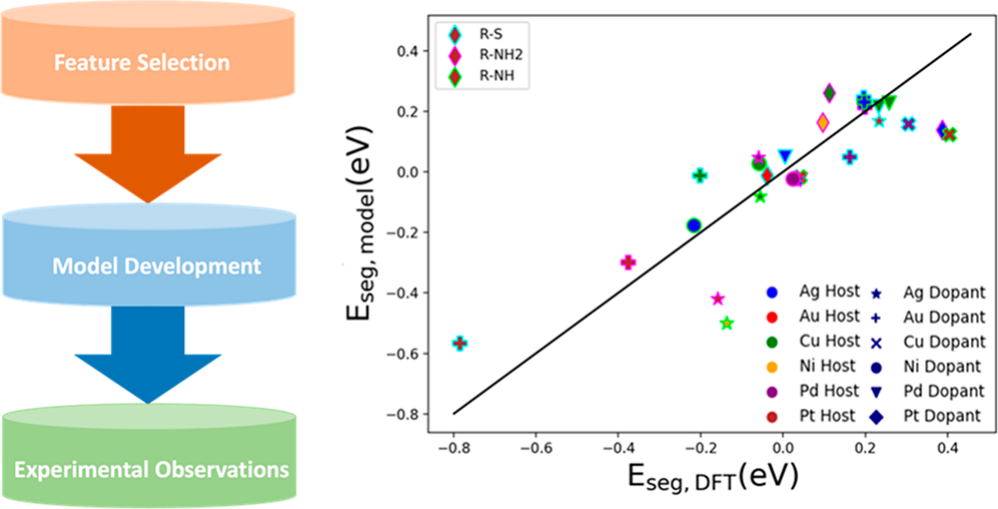

Single atom alloys (SAAs) have gained remarkable attention due to their tunable properties leading to enhanced catalytic performance, such as high activity and selectivity. The stability of SAAs is dictated by surface segregation, which can be affected by the presence of surface adsorbates. Research efforts have primarily focused on the effect of commonly found catalytic reaction intermediates, such as CO and H, on the stability of SAAs. However, there is a knowledge gap in understanding the effect of ligands from colloidal nanoparticle (NP) synthesis on surface segregation. Herein, we combine density functional theory (DFT) and machine learning to investigate the effect of thiol and amine ligands on the stability of colloidal SAAs. DFT calculations revealed rich segregation energy (*E*_seg_) data of SAAs with d^8^ (Pt, Pd, Ni) and d^9^ (Ag, Au, Cu) metals exposing (111) and (100) surfaces, in the presence and absence of ligands. Using these data, we developed an accurate four-feature neural network using a multilayer perceptron regression (NN MLP) model. The model captures the underlying physics behind surface segregation in the presence of adsorbed ligands by incorporating features representing the thermodynamic stability of metals through the bulk cohesive energy, structural effects using the coordination number of the dopant and the ligands, the binding strength of the adsorbate to the metals, strain effects using the Wigner–Seitz radius, and electronic effects through electron affinities. We found that the presence of ligands makes the thermodynamics of segregation milder compared to the bare (nonligated) SAA surfaces. Importantly, the adsorption configuration (e.g., top vs bridge) and the binding strength of the ligand to the SAA surface (e.g., amines vs thiols) play an important role in altering the *E*_seg_ trends compared to the bare surface. We also developed an accurate NN MLP model that predicts *E*_seg_ in the presence of ligands to find thermodynamically stable SAAs, leading to the rapid and efficient screening of colloidal SAAs. Our model captures several experimental observations and elucidates complex physics governing segregation at nanoscale interfaces.

## Introduction

The design of single atom active sites is desired for many catalytic reactions due to their unique physicochemical properties and potential to decrease catalyst cost.^1^ Single atom alloys (SAAs), a class of single site catalysts, consist of highly active (Pd, Pt, and Ni) dopants incorporated on the surface of less active but more selective metal hosts, typically made of d^9^ metals (Ag, Cu, and Au).^2,3^ These distinct and unique active sites have shown remarkable and improved catalytic activity against their monometallic counterparts. For example, Pei et al. demonstrated that for the semi-hydrogenation reaction, a single Pd on a Cu host produced optimal ethylene selectivity of ∼85% and 100% acetylene conversion compared to pure Cu and pure Pd.^4^

The presence of well-defined active sites results in a more rational and controlled SAA design.^2,5^ Moreover, this leads to more efficient catalysts due to their ability to alter adsorption and catalytic intermediate properties.^6^ For instance, it was found that Co_dopant_Ru_host_ and Pt_dopant_Ru_host_ exhibit enhanced catalytic activity in the production of methanol from CO_2_ due to the binding strength and charge distribution on the surface.^7^ The binding strength between the adsorbate and the surface of SAA is key to improved catalytic stability and activity. Liu et al. tested the activity of Pt_dopant_Cu_host_ compared to pure Pt for acetylene hydrogenation in the presence of CO.^8^ 50% of the reaction rate (i.e., activity) was recovered in the SAA case, while 10% was retained for the Pt nanoparticle (NP), when CO was introduced. The ∼40% difference is attributed to the binding strength of the CO to the Pt in the SAA and Pt NP, indicating that the SAA has a high tolerance to CO poisoning.^8^ Similarly, Xing et al. found that when a Pd dopant was isolated on a Cu host, a high N_2_ catalytic activity, selectivity, and stability (for ∼30 h) were attained for NO reduction in the presence of CO.^9^ These outstanding catalytic performances are due to the interaction of the adsorbate with the SAA surface under the reaction conditions. On the other hand, strong binding can lead to undesirable outcomes such as the formation of aggregates and poisoning of the catalyst. Yang indicated that the acetylene adsorption leads to surface restructuring arising from the strong binding of acetylene to the Ni_dopant_Cu_host_ and Rh_dopant_Cu_host_ SAAs, forming aggregates.^10^ Alternatively, when Pd_dopant_Cu_host_ and Pt_dopant_Cu_host_ SAAs were utilized for the same reaction, they found that the dopant remained isolated and acetylene hydrogenation to ethylene was favored.

The presence of adsorbates has a great effect on surface segregation, which is one of the key factors (a secondary factor is aggregation) dictating stability of the SAA.^2,11^ Surface segregation is defined as the thermodynamic stability of the dopant to segregate to the surface. The binding strength of the adsorbate may induce segregation of the dopant to the surface.^12^ Papanikolaou et al. have found that when platinum-group metals were doped in d^9^ metals, segregation was not likely to occur (except for PdCu). However, in the presence of CO, a reverse *E*_seg_ trend was observed due to the strong affinity of CO to platinum-group metals.^13^ It is important to note that the presence of an adsorbate may not always lead to segregation of the dopant. Wang et al. found Pt doped in Au and Ag, (111) and (100) facets, does not result in the segregation of Pt, even in the presence of H.^14^ Hence, how the presence of the adsorbate alters the segregation tendencies (i.e., thermodynamic stability of SAAs) is not straightforward to assess and requires a deep fundamental understanding of bonding interactions.

The segregation energy (*E*_seg_) of nonligated, bare surfaces (absence of any adsorbates) has been widely studied using density functional theory (DFT)^11,13,15,16^ and tight-binding theory.^17,18^ Furthermore, to accelerate the process of predicting *E*_seg_, statistical and machine learning techniques have been implemented. In our recent work, we proposed a five-feature second-order polynomial kernel ridge regression model to predict *E*_seg_ of the bare (111), (100), (110), and (210) facets on platinum group metal-based SAAs.^19^ The model incorporated as features the difference in the bulk cohesive energy divided by the coordination number of the dopant (inspired by the bond-centric model on bimetallic NPs^20^), the atomic radius of the dopant, the electronegativity of the host, the difference in the electron affinity, and the first ionization potential of the dopant.^19^ The model, which was trained on DFT results on periodic surfaces, was able to capture *E*_seg_ trends in NPs, generalizing well across different materials scales. Through this study, factors controlling host–dopant interactions that can either thermodynamically promote or hinder the segregation of the dopant were discovered. To further examine how an adsorbate affects surface segregation, there have been many studies that focused on how CO, H_2_, NO, and O_2_ alter the stability (i.e., segregation and aggregation energies) of SAAs through experimental and computational work.^8,11−14,21−23^ These adsorbates are studied either as probe molecules^13^ or as part of elementary steps for many catalytic reactions, such as hydrogenations.^16^ DFT is time-consuming (i.e., computationally expensive); hence, there is a need for a quick and accurate alternative to screen different SAA catalysts in the presence of an adsorbate. Han et al. developed a model that predicted *E*_seg_ in the presence of H through compressed-sensing data-analytics approach (SISSO), utilizing multiple DFT inputs.^16^ More recently, Sulley et al. applied machine learning techniques to determine the stability of single atom alloys in the absence and presence of CO.^24^ Although these models were able to screen through different SAAs in the presence of H and CO, an understanding of how different adsorbates, specifically ligands, affect *E*_seg_ has yet to be unraveled.

In this study, our first aim is to understand how the nature of widely used ligands, such as methylamine (H_3_C–NH_2_) and methylthiolate (H_3_C–S), affect *E*_seg_ on SAAs. −NH_2_ and −S are commonly used as ligands in noble-metal NP synthesis.^25,26^ For instance, it has been shown that the −NH_2_ and −S anchoring groups restrict growth and prevent aggregation in Ag and Au NP synthesis, respectively.^27,28^ Additionally, because the H_3_C–NH_2_ can lose H forming H_3_C–NH,^29^ we also investigate the effect of amine saturation on the adsorption configuration and *E*_seg_. We consider different SAA combinations of d^8^ (Ni, Pd, and Pt) and d^9^ (Ag, Au, and Cu) metals on low-index surfaces such as (111) and (100). Finally, with the generated DFT data, we develop a regression model using tabulated features that are able to accurately describe the surface segregation of SAAs in the presence of ligands.

## Methodology

### Density Functional Theory

The *E*_seg_ of nonligated (bare) and ligated slabs were calculated using CP2K.^30^ Exchange correlation was accounted for using the PBE functional^31^ in conjunction with Grimme’s D3 dispersion correction method.^31^ The DZVP (double-ζ valence polarized) basis set was used with the Goedecker, Teter, and Hutter (GTH) pseudopotentials at a 600 Ry cutoff.^32^ All of the calculations were spin-polarized. Self-consistent field cycles were performed with a convergence criterion of 10^–7^ hartree. Geometry relaxations were performed using the Broyden–Fletcher–Goldfarb–Shanno minimization algorithm until the forces converged to 4.0 × 10^–4^ hartree bohr^–1^.

**Figure 1:**
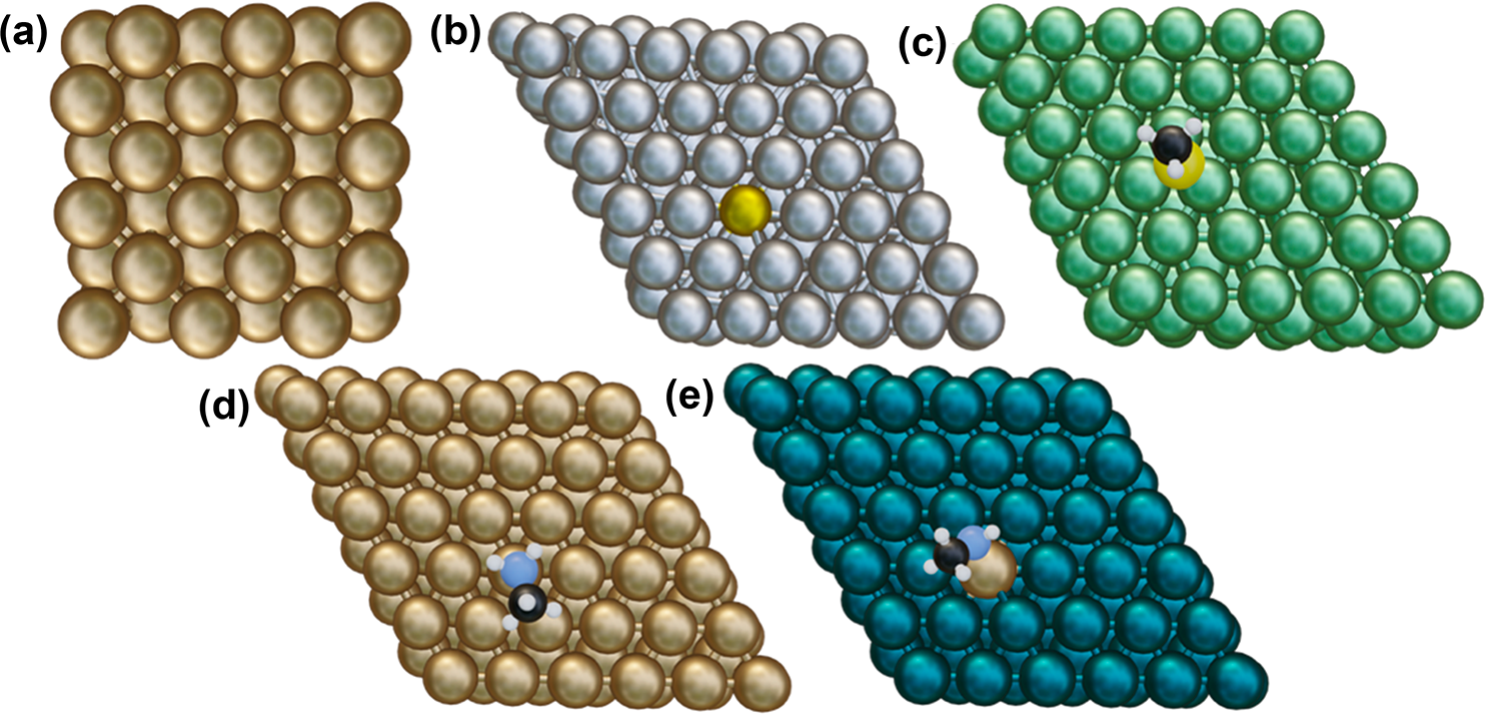
Different structures involved in *E*_seg_ calculations: (a) side view of the bulk structure, (b) top view of the dopant (Au) on the (111) host metal surface, (c) H_3_C–S hollow adsorption on a (111) surface, (d) H_3_C–NH_2_ top adsorption on a (111) surface, and (e) dopant (Cu) on a (111) metal host surface with H_3_C–NH adsorbed on the bridge position.

The bulk structure of the metals contains 108 atoms, as demonstrated in Figure 1a. The (111) and (100) slabs were modeled using a 6 × 6 × 6 cell, where the bottom three layers were fixed and the top three layers were allowed to relax, as shown in Figure 1b. The (111) surface consists of atoms with a coordination number of 9, meaning that each surface atom is coordinated with six other surface atoms and 3 additional atoms in the layer below. On the other hand, the (100) surface has a coordination number of 8, where each surface atom is bonded to 4 other surface atoms and 4 additional atoms in the layer below. Metal combinations of d^8^ (Ni, Pd, Pt) and d^9^ (Ag, Au, Cu) are considered in this study. Additionally, the adsorbates H_3_C–NH_2_, H_3_C–NH, and H_3_C–S are used. The four different cases (nonligated and 3 ligated systems) resulted in a total of 240 different systems studied in this work. It should be noted that the ligated systems (180 data points) are considered in the model development. To compute the *E*_seg_ of the bare surface, the following equation was used:^15^
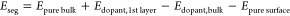
1*E*_seg_ is the segregation energy of the dopant from the bulk to the surface, and *E*_pure bulk_ and *E*_pure surface_ are the total energies of pure (monometallic) bulk and surface, respectively. The *E*_dopant,1st layer_ is the total energy of the dopant present in the first layer of the surface, and *E*_dopant,bulk_ is the total energy of the dopant present in the bulk. To account for adsorbate effects, eq 2 was used to compute *E*_seg_ in the presence of the amine and thiol ligands (*E*_seg/X_). The most stable configuration was considered in this study, i.e., hollow site for the thiolate ligand, with an exception of Au(100), where H_3_C–S prefers to form a bridge site, top site for the H_3_C–NH_2,_ and bridge site for the H_3_C–NH, as illustrated in Figure 1c–e. Thus, our data have diverse adsorption configurations due to the selection of the specific ligands.
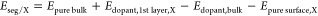
2In eq 2, *E*_seg/X_ is the segregation energy of the dopant from the bulk to the surface in the presence of an adsorbate (X). *E*_pure surface,X_ is the total energy of the monometallic surface in the presence of a ligand, as illustrated in Figures 1c and 1d. *E*_dopant,first layer,X_ is the total energy of the dopant present in the first layer in the presence of a ligand, as shown in Figure 1e. A negative *E*_seg_ value indicates that the dopant has the thermodynamic tendency to segregate to the surface, while a positive *E*_seg_ value denotes that the dopant prefers to stay in bulk.

### Machine Learning Implementation

**Figure 2:**
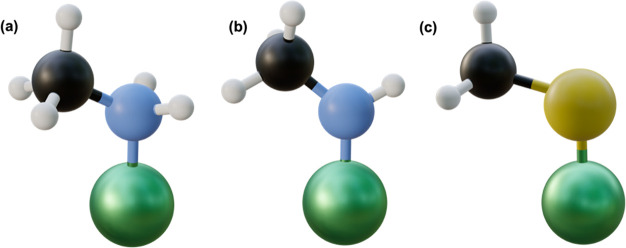
Optimized structures of a single metal atom bonded with (a) H_3_C–NH_2_ and (b) H_3_C–NH, and (c) H_3_C–S ligands. The colors represent different atoms: green is the metal atom of interest, blue is nitrogen, yellow is sulfur, black is carbon, and white is hydrogen.

We applied a supervised machine learning approach to develop an accurate *E*_seg_ regression model. Along with the binding energy of the adsorbate on a single atom (displayed in Figure 2; refer to Sections 1 and 3 of the Supporting Information for calculation details), tabulated elemental properties of the host and dopant such as the covalent radius, electronegativity, electron affinity, and first ionization potential (obtained from the Mendeleev Python package^33^) were considered as inputs. In addition to these features, the atomic radius,^34^ Wigner–Seitz radius,^35^ and bulk cohesive energy (Table S1) were also considered. The features were standardized by transforming the inputs in a manner that the distribution has a mean of 0 and a standard deviation of 1, ensuring an equal contribution of the different features. A full list of the elemental properties used in this analysis can be found in Table S2.

A 85/15% train/test split was chosen, and a 5-fold cross-validation was implemented using the training data to obtain the train and validation errors. The 15% test data was used in the final step to evaluate the accuracy of the model in predicting *E*_seg_ on unseen data using MAE and RMSE (eqs 3 and 4, respectively). For feature selection, a variable importance plot based on the random forest regression was employed to determine which features contribute more to predicting *E*_seg_ in the presence of a ligand.^36^ In our study, we use random forest regression to account for any complex (nonlinear) interactions between the features and the output.^37^
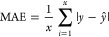
3
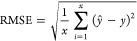
4In eqs 3 and 4, *y* is the actual output value, *ŷ* is the predicted output value, and *x* is the total number of data points. After the features were selected, the hyperparameters present in the neural network multilayer perceptron (NN MLP),^38^ kernel ridge regression (KRR),^39^ support vector regressor (SVR),^40^ random forest regressor,^41^ and extreme gradient boosting regressor (XGB)^42^ were optimized using GridSearchCV^43^ by minimizing the MAE of the validation data set. To gain a better understanding of the model’s overall performance on the data set, we evaluate the model after it is generated by using 100 different random train test splits (with different random seeds) and obtain the mean and standard deviation of the train, validation, and test set MAE. The implementation and evaluation of the models was performed using the Scikit-Learn Python package.^44^

## Results and Discussion

### DFT Calculated Segregation Trends

**Figure 3:**
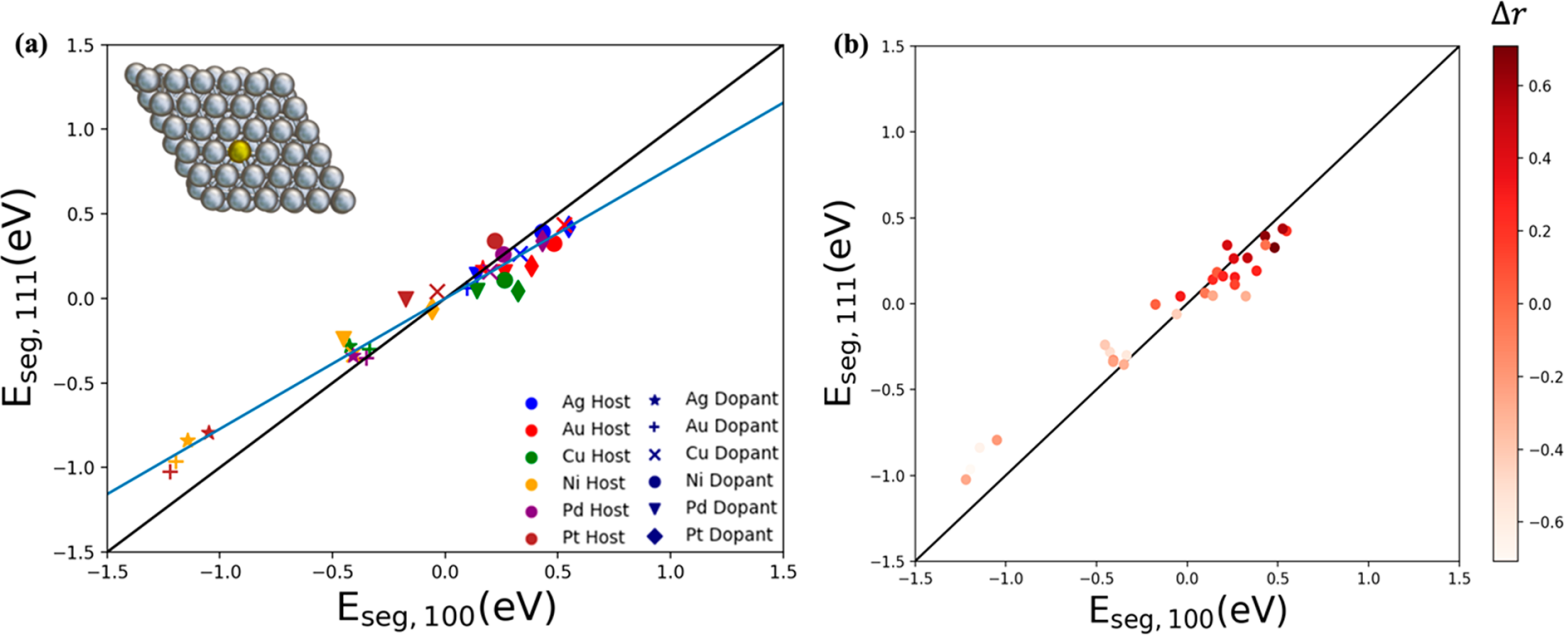
(a) Parity plot between *E*_seg,111_ and *E*_seg,100_ of d^8^- and d^9^-based SAAs in the absence of an adsorbate. Color indicates the different metal hosts, and the marker type indicates the different dopants. The inset figure demonstrates the SAA in the absence of an adsorbate. (b) Parity plot between *E*_seg,111_ and *E*_seg,100_ of d^8^- and d^9^-based SAAs in the absence of an adsorbate, with data points being colored based on the change in the radius between the metal host and dopant. A darker shade indicates that the radius of the metal host is larger than that of the dopant, while lighter shade indicates that the radius of the host is smaller than that of the dopant.

First, we compare how the DFT calculated *E*_seg_ values of bare surfaces are influenced by the different coordination environments present on the (100) and (111) surfaces (coordination number 8 on the (100) facet vs 9 on the (111) facet). In Figure 3a, we present the SAA *E*_seg_ on (111) vs the (100) facets. It can be observed that there is a linear trend, however, with a slightly changed slope from the parity (blue line vs black parity line). In cases where surface segregation was preferred (i.e., *E*_seg_ was negative), the (100) yielded more negative values than the (111) facet, indicating that the dopant had a greater thermodynamic tendency to segregate. This is because (100) has more dangling bonds than the (111) surface, causing the dopant to segregate from the bulk (high coordinated environment) to the surface (lower coordinated environment) to stabilize the system. Ag and Au metal hosts do not promote segregation of the dopant, regardless of the facet. This is because the radii of Ag and Au are larger than the radii of the d^8^ metals (shown in Figure 3b), with the atomic radius being one of the driving forces in segregation.^17^ Conversely, Ag and Au dopants are more stable on the surface of the host, regardless of the host metal. It was also found that there is a greater tendency of the dopant to segregate in the Ni-based (host) SAAs. This is because the radius of the Ni host is significantly smaller than the metal dopants considered in this study, as demonstrated in the lighter colored points in Figure 3b. Additionally, this trend is experimentally observed on AuNi systems, where Au prefers to segregate to the surface to lower the lattice strain energy arising from the change in the radius.^45^

**Figure 4:**
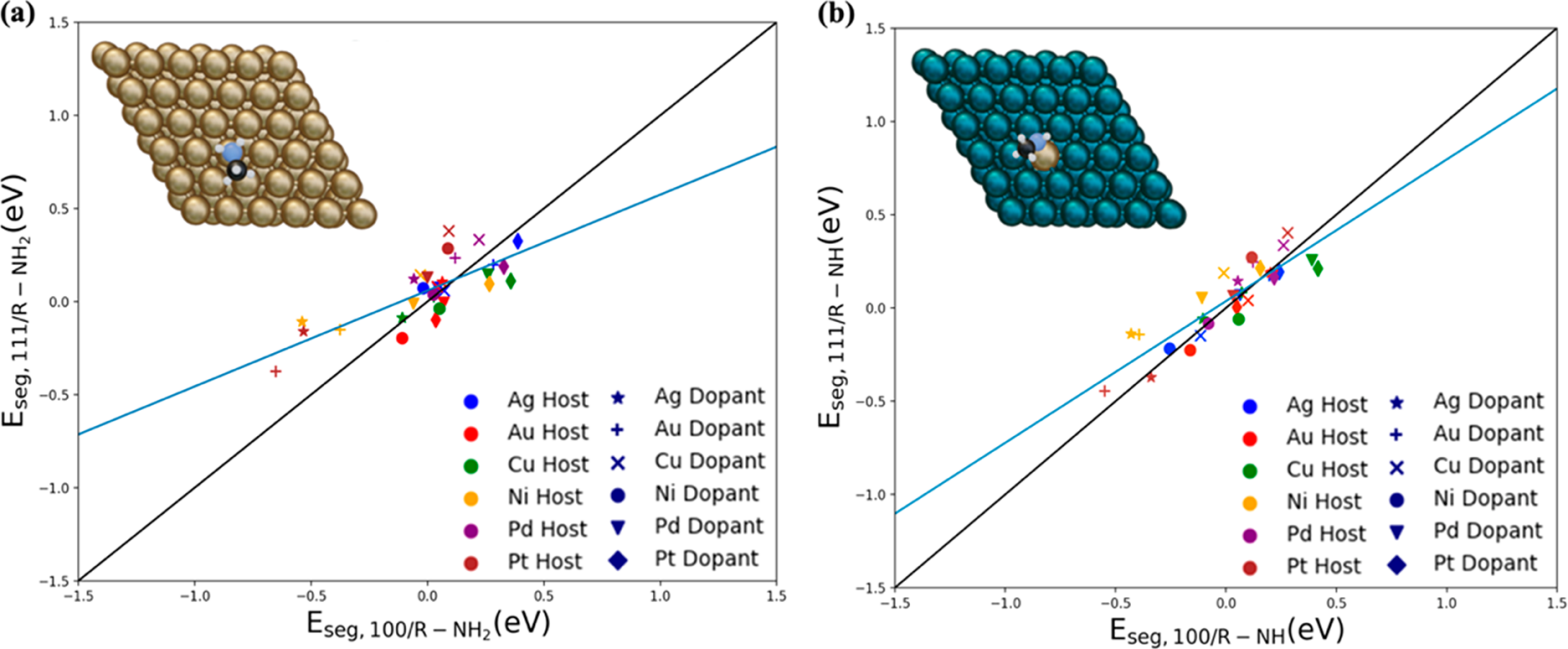
Parity plot between *E*_seg,111_ and *E*_seg,100_ of d^8^- and d^9^-based SAAs in the presence of adsorbed (a) H_3_C–NH_2_ and (b) H_3_C–NH. Colors indicate the different metal hosts, and symbols indicate the different metal dopants. The inset image demonstrates SAA in the presence of ligand.

To understand the effect of adsorbates on the *E*_seg_ trends, we first investigate the effect of the adsorption site (i.e., H_3_C–NH_2_ (top) vs H_3_C–NH (bridge) adsorption). The addition of the adsorbate has produced similar *E*_seg_ trends as the bare surface in terms of the exposed facet, meaning that the presence of the dopant on the surface is more thermodynamically preferred on the (100) than the (111) surface (shown in Figure 4). There is a wider *E*_seg_ value distribution in the bare surface compared to those in the presence of H_3_C–NH_2_ and H_3_C–NH, indicating that the presence of a ligand makes the thermodynamics of segregation milder. Interestingly, H_3_C–NH_2_ affects the slope of the *E*_seg_ data more than H_3_C–NH (compare blue lines in Figures 4a and 4b). H_3_C–NH alters the adsorption trend compared to H_3_C–NH_2_ bringing the trend back to parity, similar to the bare SAA systems, but leading to a narrower *E*_seg_ range, similar to the H_3_C–NH_2_ case. This is due to the adsorption configuration change of H_3_C–NH, which prefers to bind on a bridge site, involving two metal atoms (the dopant atom and one metal host atom), compared to the top site adsorption of H_3_C–NH_2_, which entirely involves the dopant. Thus, our results demonstrate that a top adsorption of the ligand will have a stronger effect on *E*_seg_ of a single atom compared to a bridge adsorption. It should be noted that although H_3_C–NH is a less saturated amine than H_3_C–NH_2_ and one would expect to have a stronger effect on the *E*_seg_ due to the stronger adsorption on the surface, the adsorption configuration (bridge in H_3_C–NH vs top in H_3_C–NH_2_) plays a more important role to change the slopes in Figure 4. d^8^ metals doped in d^9^ metals in the presence of H_3_C–NH_2_ and H_3_C–NH led to reverse *E*_seg_ trends. A similar effect has been reported for the same SAA combinations, when CO was introduced.^11,13^ For instance, Ni doped in Au (111) in the absence of adsorbates results in a positive *E*_seg_, meaning that the dopant prefers to reside in the bulk. In the presence of H_3_C–NH_2_ and H_3_C–NH, the *E*_seg_ behavior of Ni doped in Au (111) produced opposite (i.e., reverse) *E*_seg_ trends, promoting the dopant to the surface. On the other hand, when d^8^ metals are doped with d^9^ metals in the presence of H_3_C–NH_2_ and H_3_C–NH, an increase in the *E*_seg_ is observed. For example, in the presence of the adsorbates, Au is less likely to segregate to the Pd surface, regardless of the facet type.

**Figure 5:**
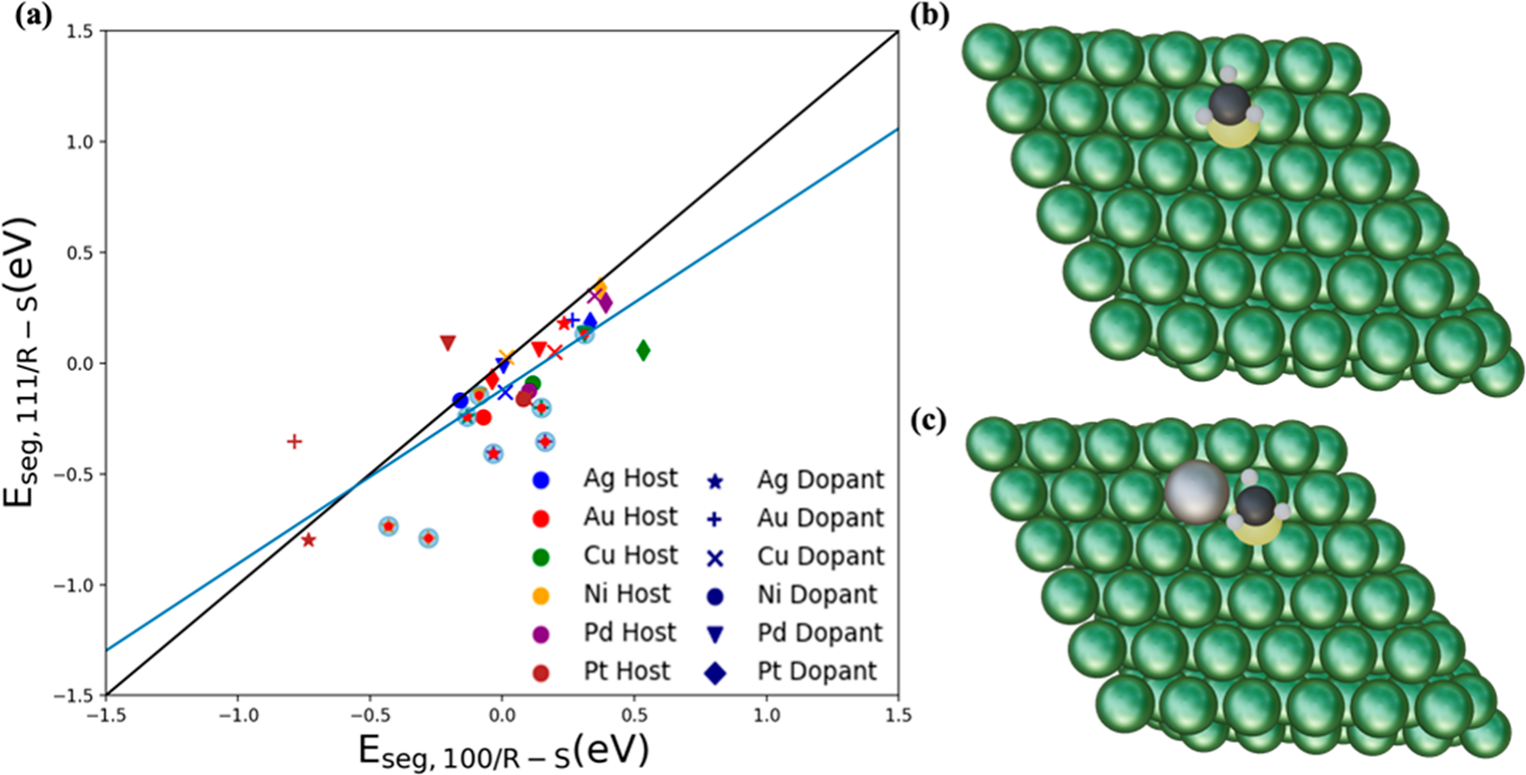
(a) Parity plot between *E*_seg,111_ and *E*_seg,100_ of d^8^- and d^9^-based SAAs in the presence of an adsorbed H_3_C–S ligand. Colors indicate the different metal hosts, and symbols indicate the different metal dopants (as in Figure 4). Top view of H_3_C–S on (b) pure Ni (111) and (c) Ag dopant on a Ni (111) surface, where the adsorbate preferentially interacts with the metal host moving away from the dopant. Silver color represents the Ag, yellow the S, black the C, and white the H atom. The blue transparent circles in (a) refer to the cases where the thiolate does not bind with the dopant but preferentially interacts with the metal host.

With regard to the H_3_C–S adsorption, the fcc-hollow adsorption is preferred for all of the metal hosts on (111) and (100) surfaces. Although the (100) facet maintained the same adsorption configuration of thiolate after the addition of the dopant, the adsorption on (111) varied. In the cases highlighted in blue in Figure 5a, the binding strength between the thiolate and host is stronger than that between the thiolate and dopant, leading to a new configuration. As a result, the thiolate–dopant bond was broken, and the thiolate formed a bond with the host metal instead during geometry optimization (shown in Figures 5b and 5c). Such a change only occurs in the (111) case due to the weaker binding of H_3_C–S on (111) compared to the (100) facet from the different surface coordination. Because of the new geometric configurations, the *E*_seg_ of H_3_C–S on the (111) surface trends changed significantly compared to the (100) surface and the other adsorbates investigated in this study (H_3_C–NH_2_ and H_3_C–NH). In the case of Cu(111)Au, the H_3_C–S adsorption changes from a hollow-site to a bridge site (still binds with the dopant and the metal host). Additionally, as a result of this adsorption deviation, a wider *E*_seg_ value distribution was found in the presence of H_3_C–S, compared to H_3_C–NH_2_ and H_3_C–NH, as illustrated in Figures 4 and 5. In the specific cases where H_3_C–S moved away from the dopant, the *E*_seg_ values are relatively similar to the bare surfaces (within 0.1 eV) due to the weak adsorbate effect on the metal dopant. This indicates how the direct coordination of the ligand to the metal dopant and the coordination environment dictate the *E*_seg_ behavior, demonstrating the complexity involved in surface segregation.

The wide range of *E*_seg_ is also attributed to the higher strength of the metal–adsorbate bond. Specifically, thiolate ligands exhibit a stronger affinity to the metals we investigated in this study (−2.29 to −4.49 eV), compared to the amine ligands, leading to noticeable deviations in the cases of thiolate–M(111). It is important to note that the binding energy of CH_3_NH_2_ to the SAA surfaces ranges from −0.57 to −1.79 eV (shown in Figure S1), leading to a shift in the segregation behavior of SAAs compared to the nonligated systems. However, the presence of an adsorbate may not always promote dopant segregation in SAAs. Wang et al. revealed that H does not always induce dopant segregation, emphasizing the significance of the metal–adsorbate bond.^14^

### Model Development

**Figure 6:**
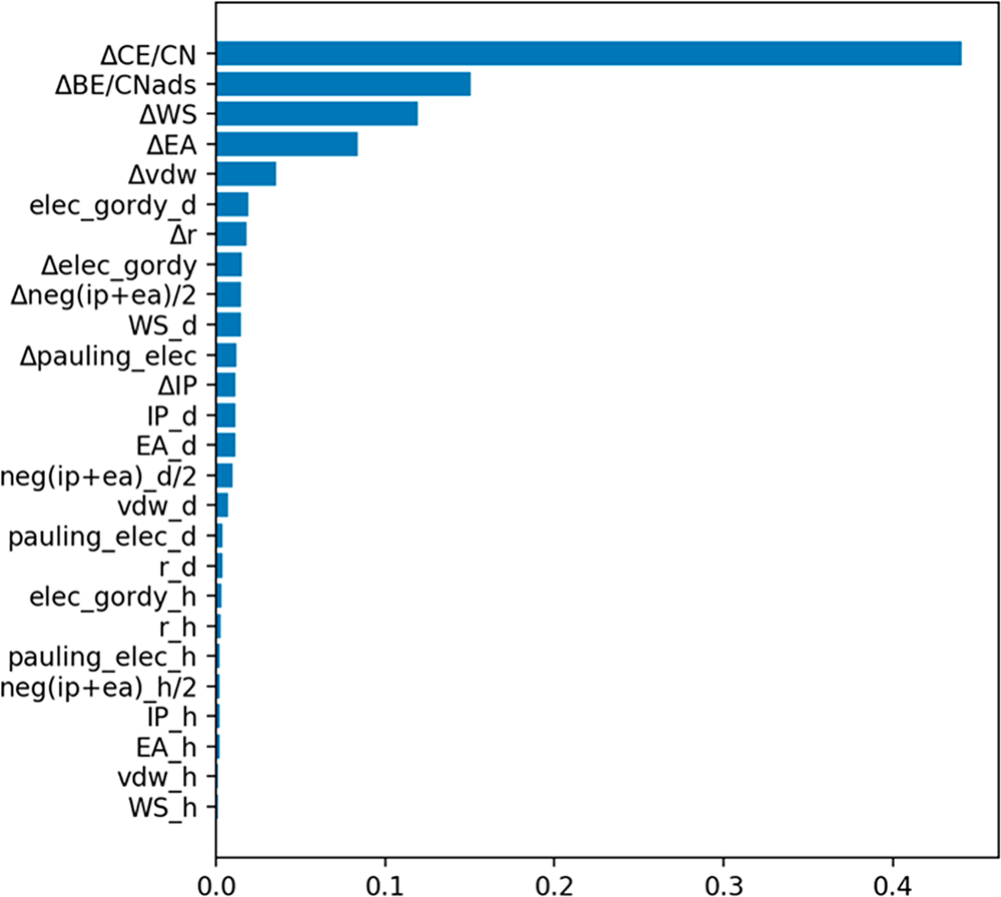
Variable importance based on random forest regression.

After gaining deep insight into how the different ligands and adsorption configurations can affect the *E*_seg_, we seek for accelerated way to screen through the different SAAs in the presence of ligands. It is infeasible to use computationally expensive DFT (i.e., time-consuming) or trial and error in experiments to screen the vast amount of possible SAA and adsorbate configurations. Supervised machine learning approaches allow us to locate optimal (i.e., with high thermodynamic stability) SAA catalysts by efficiently and accurately predicting *E*_seg_. Variable importance was employed (shown in Figure 6) to determine which variables contribute the most to predicting *E*_seg_. We also incorporated CN and CN_ads_ as separate terms (illustrated in Figure S2), and we consistently observed the retention of the same four features, indicating their importance in capturing the segregation energy in SAAs. We then implemented variance inflation factor to check for multicollinearity, which occurs when features convey redundant information. Our analysis, as depicted in Table S3, suggests that Δvdw is strongly correlated to another feature (ΔWS), evident from the significant coefficient of 15.28. Hence, we selected the top four features. Furthermore, we performed a comparative analysis between the best performing model using four features and the model’s performance when restricted to three features, with the latter showing a poor performance. These four features are the following: the difference in the bulk cohesive energy of the host and dopant divided by the coordination number of the dopant (ΔCE/CN), the difference in the binding energy of the adsorbate on a single atom of the host and dopant divided by the coordination number of the adsorbate on the surface (ΔBE/CNads; see Section 1 of the Supporting Information for details), the difference in the Wigner–Seitz radius of the host and dopant (ΔWS), and the difference in the electron affinity of the host and dopant (Δ*E*A). There is an overlap in the features between the previously developed second-order KRR model^19^ on SAA segregation on bare surfaces and the features from this analysis: ΔCE/CN, ΔEA, and a strain term such as the ΔWS, showing the transferability of these descriptors in determining the segregation behavior in the presence and absence of adsorbates. The DFT *E*_seg_ data, features, and the Python code utilized to develop the model are available free of charge on our GitHub repository (https://github.com/mpourmpakis/EsegAdsModel).

**Figure 7:**
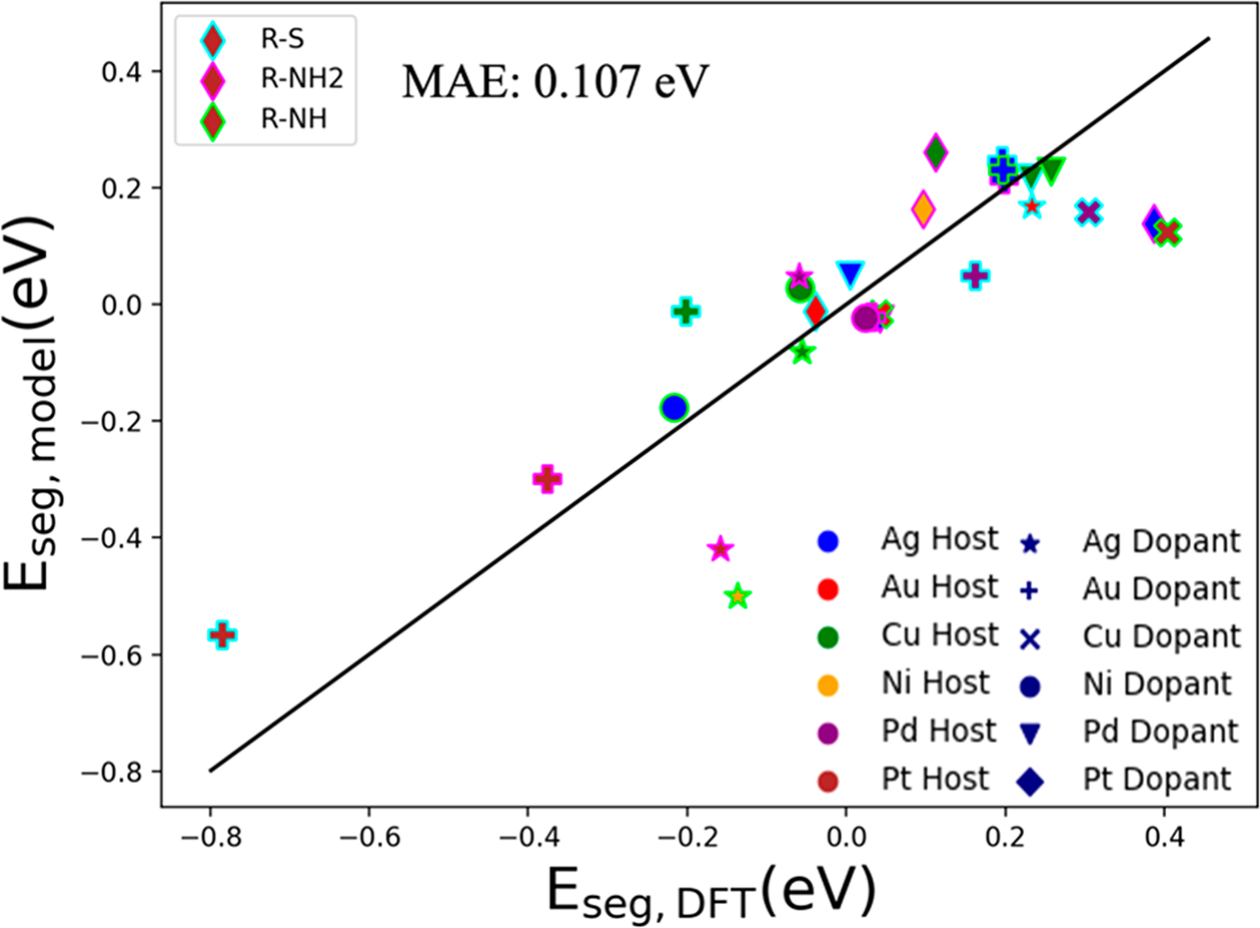
Parity plot between the NN MLP model predictions and DFT *E*_seg_ of the test set (27 data points are the test set, and 153 points are the training set). Colors indicate the different metal hosts, symbols indicate the different metal dopants, and edge color represents the adsorbate.

After the features were obtained, the hyperparameters were optimized (Table S4) and the performance of the different regression models in predicting the *E*_seg_ was compared, as demonstrated in Figure S3. Moreover, NN MLP resulted in the lowest validation MAE. We also found that the second-order KRR had comparable performance despite having fewer hyperparameters to tune; however, the KRR model misses a few cases of the *E*_seg_ behavior, specifically the antisegregation behavior, which is why NN MLP was selected. The NN MLP train, validation, and test MAEs and RMSEs were relatively similar, which denotes the model was not overfitting to the training data set, as shown in Tables S5 and S6. To better understand the model’s performance when trained on different subsets of the data, we ran 100 different train/test splits (using different random seeds) and found similar trends, as illustrated in Figure S4 and Table S7, and similar errors, further confirming that the model is not overfitting. To take a closer look at the model results on the test data set, we plot the model’s predictions against DFT *E*_seg_ (Figure 7). The model captures the *E*_seg_ trends across the different ligands, producing an MAE of 0.107 eV and an RMSE of 0.137 eV. Compared to the other adsorbates, R-NH led to the highest deviation from the parity line. Despite this deviation, our model still captures their overall segregation behavior (segregation vs thermoneutrality vs antisegregation). We also conducted a comparison with the same model, utilizing only three features. We observed a significant drop in model accuracy with a test MAE increasing to ∼0.20 eV, signifying the critical role of the top four features in capturing the *E*_seg_ trends.

The utilized features play a crucial role in the model’s performance. The four features used capture the underlying physics behind segregation in the presence of an adsorbate. The first term, ΔCE/CN, represents the thermodynamic stability of the system, while also accounting for the coordination environment of the dopant. The term is derived from the bond-centric model, used to capture the stability of metal NPs, where CE_bulk_ and CN contribute to computing the bond energies.^20,46^ The second term, ΔBE/CN_ads_, accounts for the type of ligand used and its adsorption configuration (i.e., top: CN_ads_ = 1; bridge: CN_ads_ = 2; hollow: CN_ads_ = 3). The addition of CN_ads_ is critical in capturing the different adsorption configurations that may arise, allowing for the model to distinguish between the different ligands that are considered in this study. The ΔBE is important in capturing the binding strength between the ligand and metal atom and can also capture any ligand adsorption changes that may occur on the surface of the pure host compared to the SAA (e.g., thiolate case). The third term, ΔWS, captures strain effects that are important for segregation (Figure 3b). Lastly, ΔEA qualitatively describes the electron transfer occurring between the host and dopant metals. Therefore, the four features used capture the underlying physics behind segregation in the presence of an adsorbate. This is a prime example of how critical physically relevant features are in predicting *E*_seg_ in the presence of the adsorbate.

To further validate our model’s predictions, we compare it against experimental observations (10 different experimental systems reported in Table S8). We find that the model captures the experimental observations accurately (8 out of 10 experimental observations). We do acknowledge that the model predicts thermoneutral segregation for two cases; however, our model does not consider any entropic effects, which can drive the segregation of the dopants at elevated temperatures.^2^ We note that the experimental observations are based on how dopants behave in an alloy, meaning not all the experimental results are based on highly dilute alloys but on cases where the host composition dominates the dopant concentration. This experimental validation indicates that our model shows great promise, allowing for rapid screening across SAAs in the presence of ligands. Future studies could further incorporate surface coverage and ligand size effects. Although our studies focused on one ligand adsorption on SAAs and the size of the ligand was restricted to a methyl group, the resulting segregation energy model was able to capture very complex behavior emanating from different ligands (e.g., thiols and amines), metal combinations, surface facets, and ligand adsorption configurations. We anticipate that the physical descriptors revealed in this study will play a key role in the development of more complex segregation models in the future. Our model’s applicability also extends to other adsorbates, such as CO and H, by simply calculating the ΔBE/CN_ads_ term using DFT. It is important to emphasize that this calculation is efficiently and rapidly performed, given that the systems (one metal atom bonded to one ligand) involve very few atoms.

## Conclusions

In this work, we investigated the effect of three different ligands (H_3_C–NH_2_, H_3_C–NH, and H_3_C–S) and two surface facets on the *E*_seg_ behavior of d^8^- and d^9^-based SAAs. Regardless of the adsorbate (absence or presence), in the SAA cases where segregation is favored in both facets, the (100) led to a more negative *E*_seg_ trend compared to the (111) surface. It was also found that the presence of ligands makes the thermodynamics of segregation milder compared to the trends on bare surfaces. The binding strength between the ligand and metals and the binding configuration of the ligand can lead to significant changes in the *E*_seg_ trends. These findings are critical in understanding the behavior of different adsorbates on the stability of SAAs, leading to a more efficient and informed screening of different SAA catalyst. To this goal, we leveraged machine learning techniques to predict *E*_seg_ in the presence of the three different ligands studied in this work. Based on the variable importance plot, it was determined that ΔCE/CN, ΔBE/CN_ads_, ΔEA, and ΔWS contributed the most in predicting *E*_seg_ in the presence of adsorbates. These descriptors capture the thermodynamic stability of the SAA, ligand adsorption effects, electronic modification effects, and strain effects. Multiple studies, including our own, have concluded that dopant segregation is favored when the cohesive energy of the host is larger than the cohesive energy of the dopant. Additionally, our analysis indicates that dopant segregation also depends on the coordination environment, highlighting the importance of ΔCE/CN. The second term, ΔWS, signifies that when the radius of the dopant is larger than the radius of the host, the dopant tends to be more stable on the surface. The ΔBE/CN_ads_ reveals that when the binding energy of the adsorbate to the dopant is stronger than the binding energy of the adsorbate to the host, there is a greater tendency for the dopant to segregate. Lastly, the ΔEA, describes the tendency for charge transfer between the metal and the host. These features have been previously individually shown to affect the segregation behavior, capturing the underlying physics occurring in SAA in the presence of ligands. Finally, we employed these features in different regression models and found that the NN MLP produced holistically optimal model performance compared to those of the other regression models. Our model predictions verified a series of experimental observations and elucidated important properties that can drive segregation, accelerating the controlled synthesis of SAAs.

## Supporting Information Available

Calculation details of ΔBE/CN_ads_ term; DFT calculated bulk cohesive energy (CE_bulk_); binding energy of the ligands on SAA surfaces; assessing multicollinearity using variance inflation factor; descriptors used in the feature importance analysis; extended variable importance plot; tuned hyperparameters used in regression models; parity plots of the predicted vs calculated *E*_seg_ using different regression models; MAE and RMSE scores of train, validation, and test sets of different regression models; bootstrapping analysis and related MAE scores; experimental observations from the literature against NN MLP *E*_seg_ predictions; architecture of the NN MLP; DFT electronic energy of single metal atoms, ligands, a single atom bonded to ligands, and the binding energy of the latter (PDF)
Optimized surfaces from DFT (ZIP)
