# Supplementary material for: Single Atom Alloys Segregation in the Presence of Ligands
Source: J Phys Chem C Nanomater Interfaces. 2023 Nov 13;127(46):22790–8. doi: 10.1021/acs.jpcc.3c05827 (PMC10683009; doi:10.1021/acs.jpcc.3c05827)
Supplement: Supplementary file 1 — jp3c05827_si_001.pdf [file jp3c05827_si_001.pdf]

Supporting Information

Single Atom Alloys Segregation in the Presence of Ligands

Maya Salem, Dennis J. Loevlie, and Giannis Mpourmpakis*

Department of Chemical and Petroleum Engineering, University of Pittsburgh, Pittsburgh, Pennsylvania 15261, United States

*Corresponding author. E-mail: gmpourmp@pitt.edu

1. DFT calculations for $\Delta BE/CN_{adsorbate}$ term

To calculate the $\Delta BE/CN_{ads}$ term: we take the difference between the binding energy (BE) of the adsorbate in the presence of the host and dopant. First, we compute the BE of the host or dopant by taking the difference of the electronic energy of the adsorbate binding to a single atom and electronic energy of the adsorbate in the gas phase and the host or dopant atom (Equation S1). Second, we consider the adsorption configuration of the adsorbate on the metal surface. In the case of H_3C-NH, it forms a bridge site on the surface. Hence, we can compute the average BE of the adsorbate on the metal hosts (using the BE of the adsorbate on the single atoms). It is then divided by 2 to capture the ligand adsorption coordination (Equation S2). When the dopant is incorporated, the average BE of the H_3C-NH between the host and the dopant is calculated by incorporating the BE of the H_3C-NH on a single dopant and host (Equation S3).

$$BE_X = EE_{adsorbate\text{-}metal} - EE_{adsorbate} - EE_{single\ atom\ X} \qquad (S1)$$
$$BE_{host}/CN_{ads} = (BE_{host} + BE_{host})/2 \qquad (S2)$$
$$BE_{dopant}/CN_{ads} = (BE_{host} + BE_{dopant})/2 \qquad (S3)$$

To calculate the $\Delta BE/CN_{ads}$ term, we take the difference between the Equation S2 and S3, forming Equation S4:

$$\Delta BE/CN_{ads} = BE_{host}/CN_{ads} - BE_{dopant}/CN_{ads} \qquad (S4)$$

2. Binding Energy of the Ligand to the SAA surface

The binding energy of the ligand to the SAA surface is calculated as follows:

$$BE_{SAA} = EE_{adsorbate\text{-}SAA} - EE_{adsorbate} - EE_{SAA} \qquad (S5)$$

where $EE_{adsorbate\text{-}SAA}$ and EE_{SAA} are the electronic energies of the SAA surface in the presence and absence of a ligand, respectively, and $EE_{adsorbate}$ is the electronic energy of the ligand (adsorbate).

3. Correlation between the Binding Energy of the Ligand to the SAA surface and Binding Energy of the Ligand to a Single Atom.

We observed a strong correlation between the binding energy of the ligand to the SAA surfaces and the average binding energy of the ligand to a single atom (calculated using Equation S3), as depicted in Figure S1. Expanding on this, it is important to note that determining the average binding energy of the adsorbate to the single atom requires the knowledge of the DFT-optimized surface. To address this, we assumed that in Equation S3, there will be one dopant bonded to the adsorbate. Furthermore, to account for the relative strengths, we calculated the difference between the binding energy of the adsorbate to the host atom and BE of the adsorbate to the dopant atom (Equation S4).

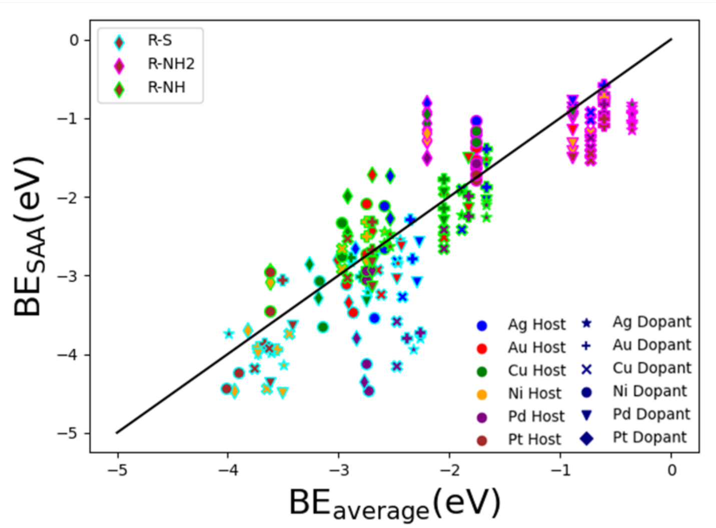

Figure S1. Parity plot between the binding energy of ligands on SAA surfaces (calculated using Equation S5) and the average binding energy of ligated atoms (calculated using Equation S3). Color indicates the different metal hosts, the marker type indicates the different metal dopants, and edge color represents the ligand.

4. List of descriptors used in the feature importance analysis

Table S1. DFT calculated CE_{bulk} used in this work.

**Metals**	**CE_{bulk} (eV/atom)**
Ag	-2.82
Au	-3.34
Cu	-3.74
Ni	-5.85
Pd	-4.17
Pt	-6.83

Table S2. Descriptors used in the feature importance analysis.

**Descriptor Name**	**Symbol**
Bulk Cohesive Energy/Coordination Number of the Dopant	CE_i/CN
Binding Energy of adsorbate on a Single Atom/Coordination Number of the Adsorbate	$\Delta BE/CN$
Atomic radius[2]	r_i
Wigner–Seitz radius[3]	WS_i
Van der Waals radius[1]	vdw_i
IPEA[1]	$-(IP_i + EA_i)/2$
Pauling Electronegativity[1]	$pauling\_elec\_i$
Gordy Electronegativity[1]	$elec\_gordy\_i$
First Ionization Potential[1]	IP_i
Electron affinity[1]	EA_i

Features with subscript i indicates that host metal property (X_h), dopant metal property (X_d), and difference between host and dopant metal (i.e. X_h - X_d) are taken into account. For example, $CE_{bulk,i}$ /CN: $CE_{bulk,h}$/CN, $CE_{bulk,d}$/CN, and $\Delta CE/CN=(CE_{bulk,h} - CE_{bulk,d})/CN$

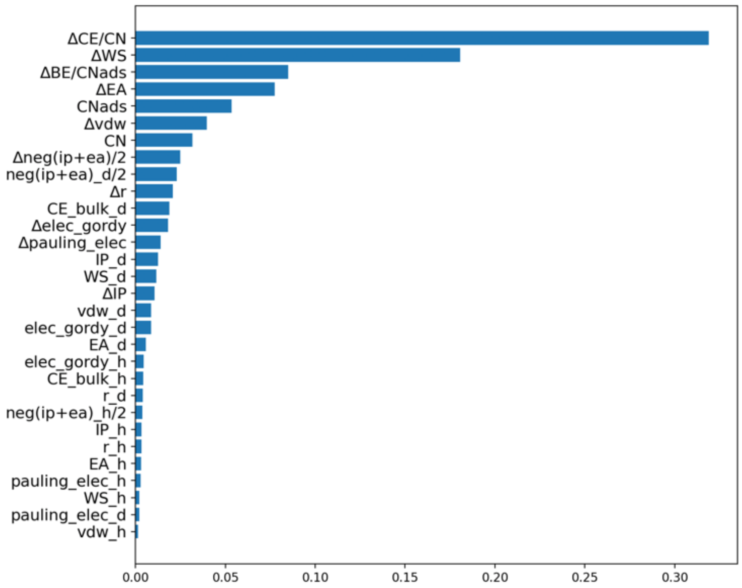

Figure S2. Variable importance (including the extended feature set) based on random forest regression.

5. Assessing Multicollinearity using Variance Inflation Factor

Table S3. Variance Inflation Factor (VIF) on the top five features.

**Feature**	**VIF value**
ΔCE/CN	1.327
$\Delta BE/CN_{ads}$	1.220
ΔWS	1.000
ΔEA	1.057
Δvdw	15.28

6. Hyperparameters used in regression models

Table S4. Hyperparameters used in the three-feature regression models (ΔCE/CN, $\Delta BE_{ads\text{-}single\ atom}/CN_{adsorbate}$, ΔWS, and ΔEA) based on the GridSearchCV results.

Model	Hyperparameter
NN MLP	**Activation**: tanh<br>**Solver**: adam<br>**Alpha**: 0.145<br>**Hidden layer**: (95,80,80,60)<br>**Epsilon**: 10^{-4}<br>**Learning_rate_init**: 0.01314<br>**n_iter_no_change:** 11<br>**tol**: 10^{-4}
KRR: 2nd Order Polynomial	**Alpha**: 0.7896842105263158<br>**Gamma**: 0. 26410526315789473
KRR: RBF	**Alpha**: 0. 47421052631578947<br>**Gamma**: 0. 26410526315789473
KRR: Laplacian	**Alpha**: 0.001<br>**Gamma**: 0.001
SVR: RBF	**C**: 1.0715071428571428<br>**Epsilon**: 0. 07235714285714286<br>**Gamma**: 0.35807142857142854
Random Forest Regressor	**Max_depth:** 4<br>**Min_samples_leaf:** 3<br>**Min_samples_split**: 10<br>**N_estimators:** 2
XGB Regressor	**Learning rate: 0.04**<br>**Max_depth:** 3<br>**Gamma**: 0.001<br>**Colsample_bytree**: 0.5

7. Predicting DFT E_{seg} using different regression models

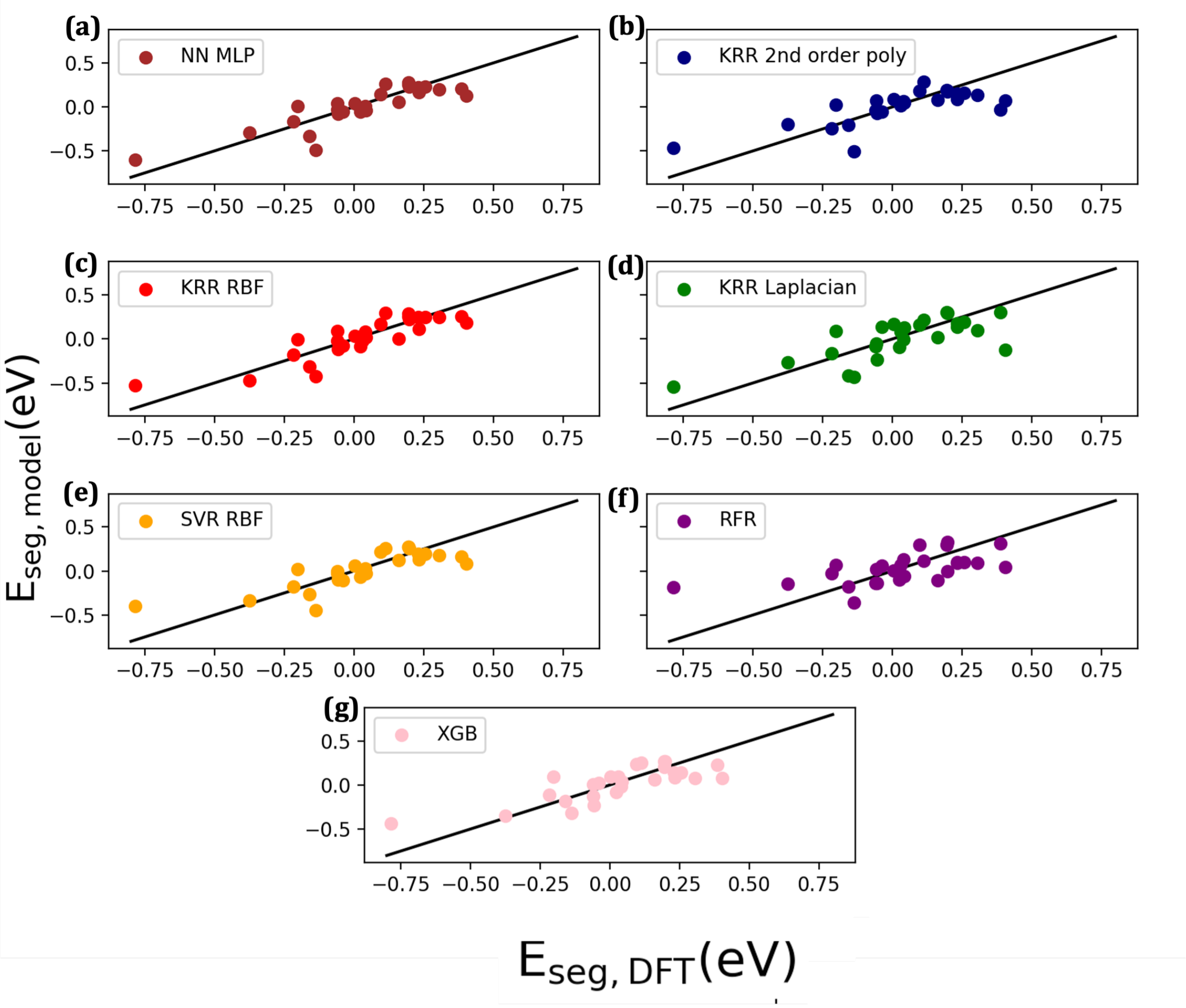

Figure S3. Parity plot between different regression models based on the test set using four features (**ΔCE/CN, $\Delta BE_{ads\text{-}single\ atom}/CN_{adsorbate}$, ΔWS, and ΔEA**) and $E_{seg,DFT}$ in the presence of $H_3C\text{-}NH_2$, $H_3C\text{-}NH$, and $H_3C\text{-}S$ data. (a) Neural Network: Multilayer perceptron, (b) Kernel Ridge Regression (KRR): 2nd order polynomial, (c) KRR: Radial Basis Function (RBF), (d) KRR: Laplacian, (e) Support Vector Regression (SVR): RBF, (f) Random Forest Regressor (RFR), and (g) Extreme Gradient Boosting Regressor (XGB).

Table S5. Different regression models and their corresponding train, test, and validation MAE.

Model	Test MAE (eV)	Validation MAE (eV)	Train MAE (eV)	Validation-Train ΔMAE (eV)
**NN MLP**	**0.107**	**0.107**	**0.094**	**0.013**
KRR: RBF	0.102	0.115	0.089	0.026
KRR: 2nd poly	0.122	0.121	0.108	0.013
KRR: Lap	0.102	0.110	0.077	0.033
SVR: RBF	0.103	0.114	0.077	0.037
Random Tree Regressor	0.125	0.126	0.109	0.017
XGB Regressor	0.112	0.109	0.080	0.028

Table S6. Different regression models and their corresponding train, test, and validation RMSE.

Model	Test RMSE (eV)	Validation RMSE (eV)	Train RMSE (eV)	Validation-Train ΔRMSE (eV)
**NN MLP**	**0.137**	**0.140**	**0.121**	**0.019**
KRR: RBF	0.134	0.151	0.118	0.033
KRR: 2nd poly	0.167	0.156	0.140	0.016
KRR: Lap	0.131	0.147	0.104	0.043
SVR: RBF	0.139	0.153	0.106	0.046
Random Tree Regressor	0.167	0.167	0.140	0.027
XGB Regressor	0.141	0.151	0.106	0.045

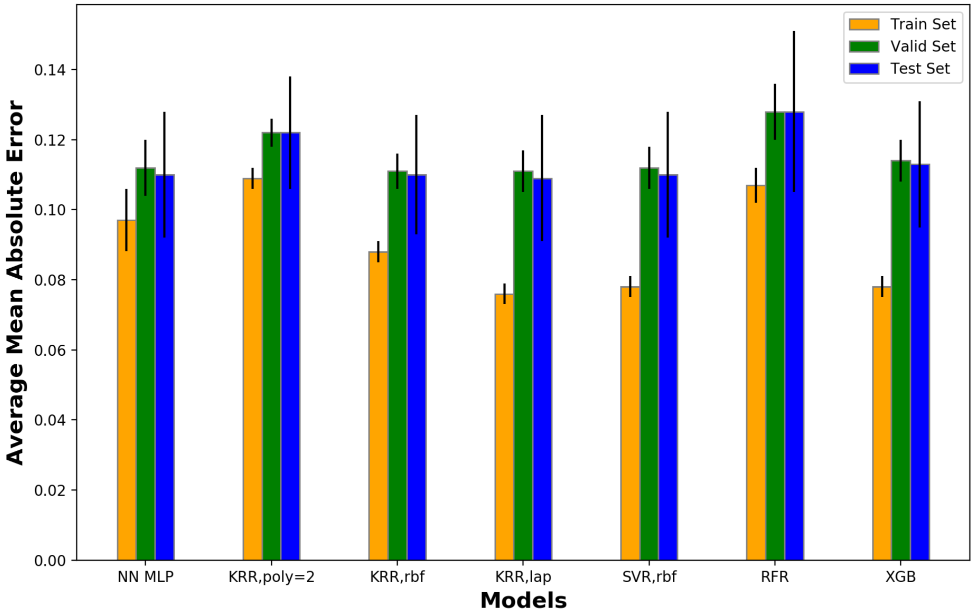

Figure S4. Average of MAE of the different models tested at 100 different train/test splits (using different random seeds). The error bars reflect the standard deviation of the different 100 train/test splits of each model.

Table S7. Different regression models and their corresponding train, test, and validation MAE with the standard deviation (run over 100 different train/test splits).

**Model**	**Test MAE (eV)**	**Validation MAE (eV)**	**Train MAE (eV)**
**NN MLP**	**0.110 ± 0.018**	**0.112± 0.008**	**0.097 ± 0.009**
KRR: RBF	0.110 ± 0.017	0.111 ± 0.005	0.088 ± 0.003
KRR: 2nd poly	0.122± 0.016	0.122 ± 0.004	0.109 ± 0.003
KRR: Lap	0.109 ± 0.018	0.111 ± 0.006	0.076 ± 0.003
SVR: RBF	0.110 ± 0.018	0.112 ± 0.006	0.078 ± 0.003
Random Tree Regressor	0.128 ± 0.023	0.128 ± 0.008	0.107 ± 0.005
XGB Regressor	0.113± 0.018	0.114± 0.006	0.078± 0.003

Table S8. Comparison between the NN MLP predictions and experimental observations.

Host	Dopant	Ligand	Experimental Observations	Predictions
Pt	Ag	amine	Ag surface[4]	Ag surface
Pt	Au	amine	Au surface[5]	Au surface
Ag	Pt	amine	Ag surface[4]	Ag surface
Pd	Ni	amine	Ni surface[6]	Thermoneutral
Cu	Au	thiol	Au surface[7]	Au surface
Au	Ag	thiol	Au surface[8]	Au surface
Ag	Au	thiol	Ag surface[8]	Ag surface
Pt	Au	thiol	Au surface[9]	Au surface
Au	Pt	thiol	Pt surface[9]	Thermoneutral
Pd	Au	thiol	Au surface[10]	Au surface

6. NN MLP architecture used in this work

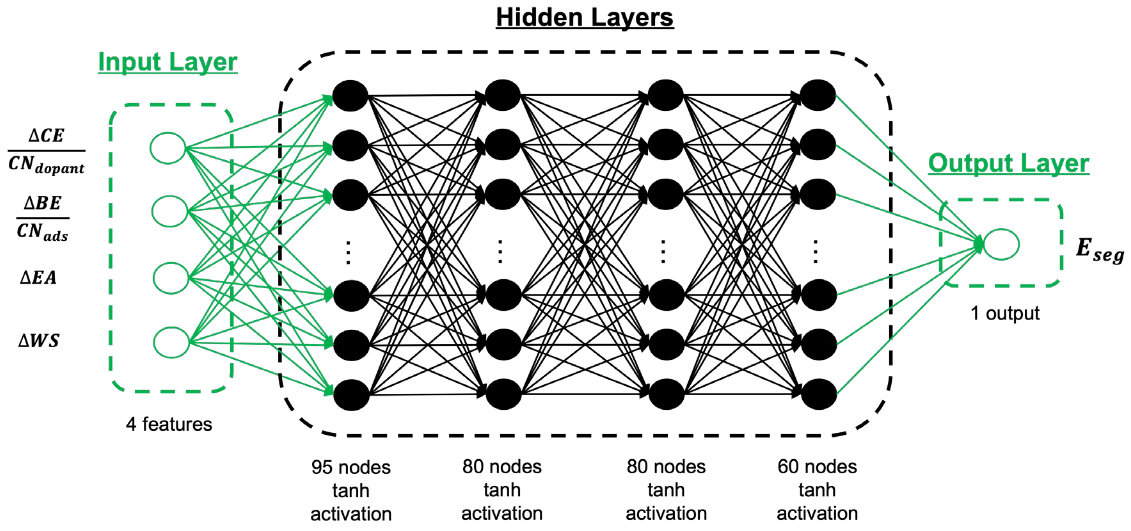

Figure S5. NN MLP architecture of the E_{seg} model.

7. DFT electronic energy data for the BE of the ligand to single atoms

Table S9. DFT electronic energy of single metal atoms, $H_3C\text{-}NH_2$, a single metal atom bonded to $H_3C\text{-}NH_2$, and the binding energy (BE) of the single atom to the $H_3C\text{-}NH_2$.

Metal	Single metal atom (Ha)	$H_3C\text{-}NH_2$ (Ha)	M-$H_3C\text{-}NH_2$ (Ha)	BE (eV)
Ag	-36.9364	-18.604372	-55.5538	-0.3533464
Au	-33.1443	-18.604372	-51.771	-0.605325
Cu	-47.9995	-18.604372	-66.6307	-0.7315446
Ni	-169.106	-18.604372	-187.775	-1.7647004
Pd	-127.12177	-18.604372	-145.759	-0.8874036
Pt	-119.9688	-18.604372	-138.654	-2.2074991

Table S10. DFT electronic energy of single metal atoms, $H_3C\text{-}NH$, a single metal atom bonded to $H_3C\text{-}NH$, and the binding energy (BE) of the single atom to the $H_3C\text{-}NH$.

.

Metal	Single metal atom (Ha)	$H_3C\text{-}NH$ (Ha)	M-$H_3C\text{-}NH$ (Ha)	BE (eV)
Ag	-36.9364	-17.943993	-54.93593	-1.5111831
Au	-33.1443	-17.943993	-51.155727	-1.8339032
Cu	-47.9995	-17.943993	-66.027408	-2.2838738
Ni	-169.106	-17.943993	-187.18503	-3.6679552
Pd	-127.12177	-17.943993	-145.13305	-1.8308724
Pt	-119.9688	-17.943993	-138.04401	-3.5706166

Table S11. DFT electronic energy of single metal atoms, H_3C-S, a single metal atom bonded to H_3C-S, and the binding energy (BE) of the single atom to the H_3C-S.

.

Metal	Single metal atom (Ha)	H_3C-S (Ha)	M-H_3C-S (Ha)	BE (eV)
Ag	-36.9364	-17.61957	-54.639677	-2.2777847
Au	-33.1443	-17.61957	-50.856907	-2.531667
Cu	-47.9995	-17.61957	-65.725453	-2.8948304
Ni	-169.106	-17.61957	-186.86929	-3.9108224
Pd	-127.12177	-17.61957	-144.82736	-2.3407246
Pt	-119.9688	-17.61957	-137.73731	-4.0528659

References

1. Mentel, L. M. Mendeleev - A Python Resource for Properties of Chemical Elements, Ions and Isotopes. 2014.
2. Clementi, E.; Raimondi, D. L.; Reinhardt, W. P. Atomic Screening Constants from Scf Functions. Ii. Atoms with 37 to 86 Electrons. *The Journal of Chemical Physics* **1967**, *47*, 1300-1307.
3. Ruban, A.; Hammer, B.; Stoltze, P.; Skriver, H. L.; Nørskov, J. K. Surface Electronic Structure and Reactivity of Transition and Noble Metals. Communication Presented at the First Francqui Colloquium, Brussels, 19–20 February 1996.1. *J Mol Catal A Chem* **1997**, *115* (3), 421–429. https://doi.org/https://doi.org/10.1016/S1381-1169(96)00348-2.
4. Nakamura, K. et al., Improvement of ORR Activity of Agpt Alloy Catalyst after Accelerated Durability Test, **2014**, *Meet. Abstr.*, 1134.
5. Suntivich, J. et al., Surface Composition Tuning of Au–Pt Bimetallic Nanoparticles for Enhanced Carbon Monoxide and Methanol Electro-oxidation*, J. Am. Chem. Soc.,* **2013**, 135, 21, 7985-7991.
6. Costa, N.J.S. et al., Organometallic Preparation of Ni, Pd, and NiPd Nanoparticles for the Design of Supported Nanocatalysts, *ACS Catal.,* **2014**, 4,6,1735-1742.
7. Bracey, C.L. et al., Application of copper–gold alloys in catalysis: current status and future perspectives, *Chem. Soc. Rev.*, **2009**, 38, 2231-2243.
8. Li, Z.Y. et al., Structures and optical properties of 4–5 nm bimetallic AgAu nanoparticles, *Faraday Discussions*, **2007**, 138, 363-373.
9. Torres-Pacheco, L.J. et al., Sorbitol electro-oxidation reaction on sub<10 nm PtAu bimetallic nanoparticles, *Electrochimica Acta*, **2020**, 353, 136593.
10. Knecht, M.R. et al., Structural Rearrangement of Bimetallic Alloy PdAu Nanoparticles within Dendrimer Templates to Yield Core/Shell Configurations, *Chem. Mater.*, **2008**, 20, 3, 1019-1028.
